# 骨髓增生异常综合征白血病转化危险因素研究

**DOI:** 10.3760/cma.j.issn.0253-2727.2022.10.004

**Published:** 2022-10

**Authors:** 颂扬 赵, 泽锋 徐, 铁军 秦, 士强 曲, 承文 李, 玉娇 贾, 丽娟 潘, 冰 李, 清妍 高, 蒙 焦, 慧君 黄, 志坚 肖

**Affiliations:** 中国医学科学院血液病医院（中国医学科学院血液学研究所），实验血液学国家重点实验室，国家血液系统疾病临床医学研究中心，细胞生态海河实验室，天津 300020 State Key Laboratory of Experimental Hematology, National Clinical Research Center for Blood Diseases, Haihe Laboratory of Cell Ecosystem, Institute of Hematology & Blood Diseases Hospital, Chinese Academy of Medical Sciences & Peking Union Medical College, Tianjin 300020, China

**Keywords:** 骨髓增生异常综合征, 白血病转化, 危险因素, 克隆演化, Myelodysplastic syndromes, Leukemia transformation, Risk factors, Clonal evolution

## Abstract

**目的:**

探讨骨髓增生异常综合征（MDS）白血病转化（LT）的危险因素。

**方法:**

收集2012年 1月至2020年12月中国医学科学院血液病医院MDS诊疗中心确诊、具有完整的临床资料且进行LT情况随访的320例初诊的原发性MDS患者，回顾性分析发生LT的MDS患者初诊时临床和分子学特征以及MDS患者发生LT的危险因素。

**结果:**

中位随访13.6（0.4～107.3）个月，随访期间共有75例（23.4％）MDS患者发生LT（LT组）。与未发生LT患者（未发生LT组）相比，LT组患者年龄更大（60岁对48岁，*P*<0.001）、骨髓原始细胞比例更高（7.0％对2.5％，*P*<0.001）。两组患者中骨髓纤维化（MF-2/3级比例）（13.9％对6.5％，*P*＝0.046）、WHO分型诊断构成比（*P*<0.001）、修订版国际预后积分系统（IPSS-R）分组（*P*<0.001）和IPSS-R细胞遗传学分组（*P*＝0.001）比较，差异均有统计学意义。初诊时，LT组和未发生LT组患者中位基因突变数目分别为1（1，3）个、1（0，2）个（*P*＝0.003）。LT组TP53（*P*＝0.034）、DNMT3A（*P*＝0.026）、NRAS（*P*＝0.027）、NPM1（*P*＝0.017）基因突变率显著高于未发生LT组。6例患者初诊和LT时两次二代测序结果比较，LT时患者中位基因突变数目增多［2（0～8）个对0.5（0～4）个］，中位等位基因突变频率（VAF）较初诊时明显增加。多因素Cox分析显示，骨髓原始细胞比例［以<5％为参照，5％～10％的*HR*＝4.587，95％*CI* 2.214～9.504，*P*<0.001；>10％的*HR*＝9.352，95％*CI* 4.049～21.600，*P*<0.001］，IPSS-R细胞遗传学分组差和极差（*HR*＝2.603，95％*CI* 1.229～5.511，*P*＝0.012）、DNMT3A突变（*HR*＝4.507，95％*CI* 1.889～10.753，*P*＝0.001）、NPM1突变（*HR*＝3.341，95％*CI* 1.164～9.591，*P*＝0.025）是MDS发生LT的独立危险因素。

**结论:**

骨髓原始细胞比例高、IPSS-R细胞遗传学分组差和极差、DNMT3A突变、NPM1突变是MDS发生LT的独立危险因素。

## Abstract (en)

**Objective:**

To explore the risk factors in leukemia transformation（LT）in those with myelodysplastic syndromes（MDS）.

**Methods:**

From January 2012 to December 2020,data on 320 patients with newly diagnosed primary MDS were gathered from the MDS center. The clinical features and molecular characteristics are explored. Additionally, a retrospective analysis of risk factors for the development of acute leukemia from MDS was done.

**Results:**

The median follow-up was13.6（0.4–107.3）months. 23.4％（75/320）of the MDS patients had LT group. Significant differences between the LT group and non-LT group can be seen in age（*P*<0.001）, bone marrow blast percentage（*P*<0.001）, bone marrow fibrosis（*P*＝0.046）, WHO classification（*P*<0.001）, IPSS-R（*P*<0.001）and IPSS-R karyotype group（*P*＝0.001）. The median number of mutation of LT group was 1（1, 3）, that in non-LT group was 1（0, 2）,which had a statistical difference（*P*＝0.003）.At the time of the initial diagnosis of MDS, the LT group had higher rates of the TP53 mutation（*P*＝0.034）, DNMT3A mutation（*P*＝0.026）, NRAS mutation（*P*＝0.027）and NPM1 mutation（*P*＝0.017）. Compared with the mutations at first diagnosis and LT of six patients, the number of mutations increased and the variant allele frequencies（VAF）increased significantly in LT patients. Higher bone marrow blast percentage（Refer to <5％, 5％–10％: *HR*＝4.587, 95％*CI* 2.214 to 9.504, *P*<0.001, >10％: *HR*＝9.352, 95％*CI* 4.049 to 21.600, *P*<0.001）, IPSS-R cytogenetic risk groups（*HR*＝2.603, 95％*CI* 1.229–5.511, *P*＝0.012）, DNMT3A mutation（*HR*＝4.507, 95％*CI* 1.889–10.753, *P*＝0.001）, and NPM1 mutation（*HR*＝3.341, 95％*CI* 1.164–9.591, *P*＝0.025）were all independently associated with LT in MDS patients, according to results of multivariate Cox regression.

**Conclusion:**

Bone marrow blast percentage, IPSS-R cytogenetic risk groups, DNMT3A mutation, and NPM1 mutation are independent risk factors in LT for MDS patients.

骨髓增生异常综合征（MDS）是一组以外周血血细胞减少、骨髓一系或多系发育异常、高风险向急性白血病转化为特征的异质性髓系克隆性疾病[1]–[2]。白血病转化（LT）的MDS患者对传统化疗反应差，预后不佳，如果能早期识别可能发生LT的高风险MDS患者，则可以及早启动治疗，改善患者预后[3]。临床上多应用修订的国际预后积分系统（IPSS-R）来评估MDS初诊患者LT风险和预后，随着二代测序技术的发展，突变基因对LT的影响也逐渐受到重视，但目前还未形成一套新的公认的模型预测MDS发生的LT风险。本研究我们通过回顾性研究分析我院发生LT的初诊MDS患者的临床特征和分子学特征，探讨MDS LT的危险因素。

## 病例与方法

1. 病例：2012年1月至2020年12月中国医学科学院血液病医院MDS诊疗中心确诊、具有完整的临床资料和进行LT情况随访的320例初诊原发性MDS患者纳入本研究。

2. 染色体核型分析：骨髓细胞经过24 h培养后，收集细胞并常规制片、R显带，根据《人类细胞遗传学国际命名体制（ISCN2013）》描述核型异常，按IPSS-R细胞遗传学分组标准[4]进行分组。

3. 靶向二代测序：收集患者骨髓，分离单个核细胞，常规提取DNA并制备DNA全基因组文库，PCR引物扩增目的基因组（包含112个血液肿瘤相关基因），将目标区域的DNA富集后，在Ion Torrent测序平台进行测序。测序后利用CCDS、人类基因组数据库（HG19）、dsSNP（v138）、1000 genomes、COSMIC、PolyPhen-2等数据库对原始数据进行生物信息学分析，筛选出致病性基因突变位点。具体操作方法详见本课题组已发表的文献[5]–[7]。

4. 克隆时序分析：利用等位基因突变频率（VAF）对携带两种及以上突变的患者进行突变时序分析。VAF值显著高（VAF值相差≥10％）的突变基因为主克隆，显著低的突变为亚克隆，当不同基因突变的VAF值无显著差异时，均判定为主克隆。采用LT时基因突变VAF值/MDS初诊时VAF值的比值分析基因突变负荷变化，比值>1.2和<0.8分别定义突变负荷增加或减少，比值在0.8～1.2则定义为突变负荷稳定[8]–[9]。

5. 随访：所有病例随访至白血病转化、造血干细胞移植、死亡或2021年3月1日，随访资料来源于门诊病历、住院病历及电话随访记录，中位随访13.6（0.4～107.3）个月。对随访期间死亡病例，根据病历记录或电话联系家属确认。LT时间定义为MDS确诊至MDS转化为急性白血病时间。

6. 统计学处理：统计学软件采用SPSS 25.0。分类变量以频率表示，组间比较行卡方检验或Fisher精确概率法；不符合正态分布的连续变量以“中位数（范围）”表示，组间比较采用Mann-Whitney *U*检验，配对非正态样本采用 Kruskal-Wallis *H*检验。生存分析应用Kaplan-Meier法，组间比较利用Log-rank法。应用Cox回归模型进行单因素和多因素分析探讨MDS LT危险因素。*P*<0.05表示差异有统计学意义。

## 结果

一、患者的临床特征和LT情况

320例MDS患者中男198例（61.9％），女122例（38.1％），中位年龄51（15～83）岁。依据WHO 2016诊断标准：MDS伴单系发育异常（MDS-SLD）20例（6.3％）、MDS伴多系发育异常（MDS-MLD）123例（38.4％）、MDS伴环形铁粒幼红细胞（MDS-RS）18例（5.6％）、MDS伴原始细胞增多-1（MDS-EB-1）75例（23.4％）、MDS伴原始细胞增多-2（MDS-EB-2）66例（20.6％）、MDS伴5q− 3例（0.9％），MDS未分类（MDS-U）15例（4.7％）。

284例（88.8％）患者确诊时具有可分析的染色体核型结果，IPSS-R分组：极低危11例（3.9％）、低危67例（23.6％）、中危99例（34.9％）、高危58例（20.4％）、极高危49例（17.3％）。

264例（82.5％）患者可追踪到治疗方案，32例（12.1％）仅单纯支持治疗，48例（18.2％）接受免疫抑制药物或免疫调节药物治疗，71例（26.9％）接受去甲基化药物治疗，86例（32.6％）接受异基因造血干细胞移植，12例（4.5％）接受CAG/HAG（阿克拉霉素/高三尖杉酯碱+阿糖胞苷+G-CSF）方案化疗，15例（5.7％）接受中医中药治疗。

中位随访13.6（0.4～107.3）个月，320例MDS患者中75例（23.4％）发生LT。IPSS-R极低危组患者例数较少未纳入生存分析，IPSS-R低危组、中危组、高危组、极高危组的中位无白血病转化时间依次为：未到达、未到达、24.4（95％*CI* 10.4～38.4）个月、12.0（95％*CI* 8.4～15.5）个月（*P*<0.001）。

二、LT患者的临床特征

发生LT患者（75例）中位年龄为60（20～77）岁，显著高于未发生LT组（245例）的48（15～83）岁，差异有统计学意义（*z*＝−4.296，*P*<0.001）。两组患者骨髓原始细胞比例中位数［7.0％（0～19.0％）对2.5％（0～18.5％），*P*<0.001］、骨髓纤维化（MF-2/3级比例为13.9％对6.5％，*P*＝0.046）、WHO分型诊断构成比（*P*<0.001）、IPSS-R分组（*P*<0.001）和IPSS-R细胞遗传学分组（*P*＝0.001）差异均有统计学意义。而性别，外周血HGB、中性粒细胞绝对值、PLT，LDH水平，血细胞减少系别数两组差异均无统计学意义（表1）。

**表1:** 骨髓增生异常综合征（MDS）白血病转化（LT）患者的临床特征

临床特征	LT组（75例）	未发生LT组（245例）	统计量	*P*值
年龄[岁，*M*（范围）]	60（20~77）	48（15~83）	*ｚ*=−4.296	<0.001
男性[例（%）]	49（65.3）	149（60.8）	*χ^2^*=0.497	0.481
HGB[g/L，*M*（范围）]	80（43~147）	81（41~150）	*ｚ*=−1.770	0.860
ANC[×10^9^/L，*M*（范围）]	1.14（0.18~13.18）	1.12（0.14~11.63）	*ｚ*=−0.339	0.735
PLT[×10^9^/L，*M*（范围）]	59（2~514）	62（6~553）	*ｚ*=−0.486	0.627
外周血细胞减少系数[例（%）]			*χ^2^*=3.378	0.066
0~1系	24（32.0）	53（21.6）		
2~3系	51（68.0）	192（78.4）		
WHO分型（2016）[例（%）]				<0.001
MDS-SLD	2（2.7）	18（7.3）		
MDS-MLD	14（18.7）	109（44.5）		
MDS-RS	0（0）	18（7.3）		
MDS-EB-1	31（41.3）	44（18.0）		
MDS-EB-2	26（34.7）	40（16.3）		
MDS伴5q−	1（1.3）	2（0.8）		
MDS-U	1（1.3）	14（5.7）		
骨髓原始细胞比例[%，*M*（范围）]	7.0（0~19.0）	2.5（0~18.5）	*ｚ*=−5.938	<0.001
IPSS-R细胞遗传学分组[例（%）]				0.001
极好	0（0）	5（2.3）		
好	35（49.3）	135（63.4）		
中等	15（21.1）	45（21.1）		
差	6（8.5）	18（8.5）		
极差	15（21.1）	10（4.7）		
IPSS-R预后分组[例（%）]				<0.001
极低危	0（0）	11（5.2）		
低危	10（14.1）	57（26.8）		
中危	16（22.5）	83（39.0）		
高危	20（28.2）	38（17.8）		
极高危	25（35.2）	24（11.3）		
LDH水平[U/L，*M*（范围）]	212.5（66~938）	205.2（96~843）	*ｚ*=−0.545	0.586
骨髓纤维化分级[例（%）]			*χ^2^*=3.966	0.046
MF-0/1级	62（86.1）	216（93.5）		
MF-2/3级	10（13.9）	15（6.5）		
基因突变[例（%）]				
U2AF1	11（17.5）	46（20.6）	*χ^2^*=0.309	0.578
DNMT3A	9（14.3）	12（5.4）		0.026
TP53	8（12.7）	10（4.5）		0.034
ASXL1	7（11.1）	26（11.7）	*χ^2^*=0.014	0.904
RUNX1	6（9.5）	17（7.6）	*χ^2^*=0.240	0.624
NPM1	6（9.5）	5（2.2）		0.017
NRAS	5（7.9）	4（1.8）		0.027
SF3B1	4（6.3）	24（10.8）	*χ^2^*=1.083	0.298
TET2	4（6.3）	10（4.5）		0.517

注：ANC：中性粒细胞绝对计数；MDS-SLD：MDS伴单系发育异常；MDS-MLD：MDS伴多系发育异常；MDS-RS：MDS伴环形铁粒幼红细胞；MDS-EB-1：MDS伴原始细胞增多-1；MDS-EB-2：MDS伴原始细胞增多-2；MDS-U：MDS，不能分类型；IPSS-R：修订版国际预后积分系统

三、LT患者的分子学特征

二代测序结果显示LT组（63例）患者中位基因突变数目为1（1，3）个，未发生LT组为1（0，2）个，差异有统计学意义（*P*＝0.003）（图1）。69.6％（199/286）的患者初诊时检出至少1个基因突变，常见的基因突变（突变检出率≥5％）依次是U2AF1（57例，19.9％）、ASXL1（33例，11.5％）、SF3B1（28例，9.8％）、RUNX1（23例，8.0％）、DNMT3A（21例，7.3％）和TP53（18例，6.3％）。LT组患者中发生频率较高（突变检出率≥5％）的突变依次是：U2AF1（11例，17.5％）、DNMT3A（9例，14.3％）、TP53（8例，12.7％）、ASXL1（7例，11.1％）、RUNX1（6例，9.5％）、NPM1（6例，9.5％）、NRAS（5例，7.9％）、SF3B1（4例，6.3％）、TET2（4例，6.3％）。两组基因突变谱详见图2。LT组TP53（*P*＝0.034）、DNMT3A（*P*＝0.026）、NRAS（*P*＝0.027）、NPM1（*P*＝0.017）基因突变率显著高于未发生LT组。LT组常见的基因突变（U2AF1、DNMT3A、TP53、ASXL1、RUNX1、NPM1、NRAS、SF3B1、TET2）的VAF值与未发生LT组比较差异均无统计学意义（*P*值均>0.05）。

**图1:**
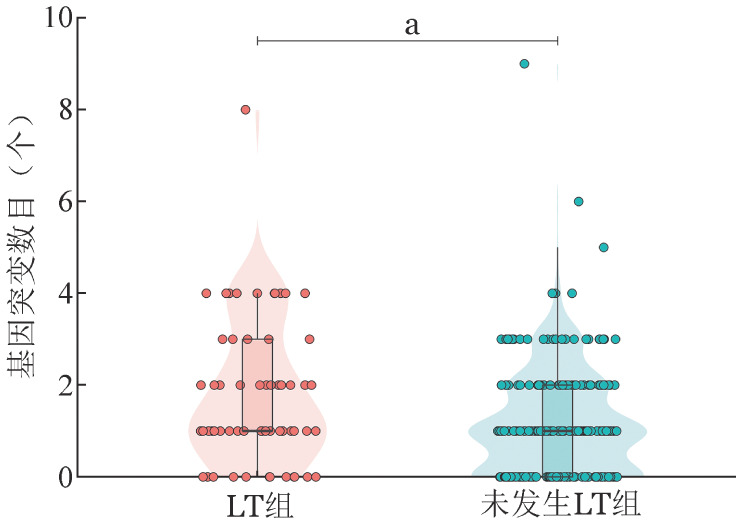
发生LT（LT组）和未发生LT（未发生LT组）MDS患者初诊时基因突变数目比较（^a^*P*<0.05） LT：白血病转化；MDS：骨髓增生异常综合征

**图2:**
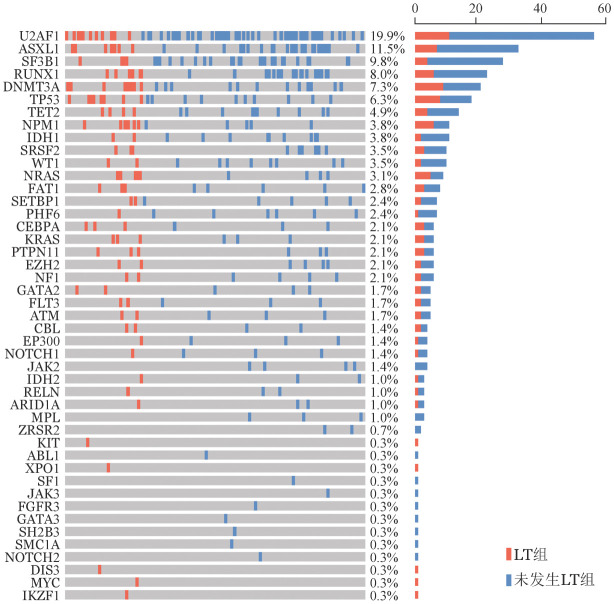
发生LT（LT组）和未发生LT（未发生LT组）MDS患者基因突变谱 LT：白血病转化；MDS：骨髓增生异常综合征

6例患者具有初诊和LT时两次二代测序结果（图3），4例患者获得至少1个新的基因突变，初诊时6例患者中位基因突变数目0.5（0～4）个，LT时中位基因突变数目2（0～8）个，基因突变数目有增多的趋势。初诊与LT时基因突变VAF值比较，LT时多数基因VAF较初诊时明显增加。因具有连续标本的患者例数过少未对患者基因演化进行统计学分析。

**图3:**
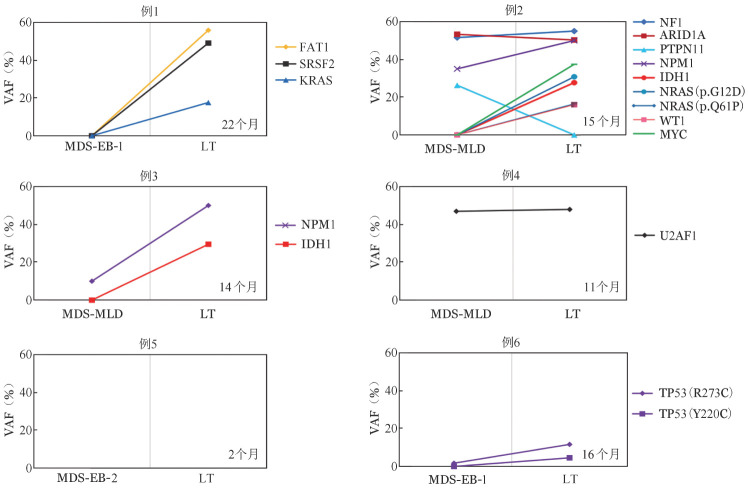
6例发生LT的MDS患者在MDS初诊和LT时二代测序结果 黑色：RNA剪接基因突变；蓝色：信号传导相关突变；红色：表观遗传学相关突变；绿色：转录因子；紫色：细胞周期与凋亡相关突变；黄色：其他突变。MDS：骨髓增生异常综合征；LT：白血病转化；VAF：等位基因突变频率

四、影响MDS发生LT的危险因素

单因素分析显示初诊时年龄≥60岁（*P*＝0.002）、骨髓原始细胞比例5％～10％或>10％（*P*<0.001）、IPSS-R细胞遗传学分组差和极差（*P*<0.001）、MF-2/3级（*P*＝0.001）、基因突变数目≥3个（*P*＝0.001）、DNMT3A突变（*P*＝0.001）、TP53突变（*P*＝0.001）、NRAS突变（*P*＝0.012）、NPM1突变（*P*<0.001）是MDS发生LT的危险因素。将单因素分析*P*<0.1的因素纳入Cox多因素分析，结果显示骨髓原始细胞比例5％～10％与>10％、IPSS-R细胞遗传学分组差和极差、DNMT3A突变、NPM1突变是MDS发生LT的独立危险因素（表2）。

**表2:** 影响骨髓增生异常综合征（MDS）发生白血病转化（LT）的单因素与多因素分析

因素	单因素分析		多因素分析	
	*HR*（*95%CI*）	*P*值	*HR*（*95%CI*）	*P*值
年龄≥60岁	2.043（1.297~3.218）	0.002	1.168（0.617~2.212）	0.634
男性	1.320（0.820~2.125）	0.252		
HGB<100 g/L	1.227（0.722~2.084）	0.450		
ANC<0.8×10^9^/L	1.318（0.810~2.142）	0.266		
PLT≥100×10^9^/L	0.935（0.578~1.512）	0.785		
外周血细胞2~3系减少	0.813（0.500~1.322）	0.405		
骨髓原始细胞比例				
<5％	参照		参照	
5％~10％	6.721（3.854~11.720）	<0.001	4.587(2.214~9.504）	<0.001
>10％	10.641（5.462~20.731）	<0.001	9.352（4.049~21.600）	<0.001
IPSS-R细胞遗传学差和极差	3.836（2.278~6.459）	<0.001	2.603（1.229~5.511）	0.012
LDH>247 U/L	1.370（0.836~2.245）	0.212		
MF-2/3级	3.106（1.584~6.091）	0.001	2.461（0.934~6.482）	0.068
基因突变数目≥3个	2.662（1.520~4.663）	0.001	1.359（0.655~2.819）	0.409
U2AF1突变	0.970（0.505~1.864）	0.927		
DNMT3A突变	3.199（1.575~6.497）	0.001	4.507（1.889~10.753）	0.001
TP53突变	3.487（1.651~7.365）	0.001	1.714（0.668~4.397）	0.263
ASXL1突变	0.919（0.418~2.016）	0.832		
RUNX1突变	1.411（0.608~3.275）	0.423		
NPM1突变	4.676（1.996~10.957）	<0.001	3.341（1.164~9.591）	0.025
NRAS突变	3.260（1.303~8.153）	0.012	1.792（0.564~5.695）	0.323
SF3B1突变	0.561（0.204~1.546）	0.264		
TET2突变	2.307（0.834~6.379）	0.107		

注：ANC：中性粒细胞绝对计数；IPSS-R：修订版国际预后积分系统；MF：骨髓纤维化

## 讨论

如何识别可能发生LT的MDS患者一直是临床难题，目前临床中多应用IPSS-R评估MDS发生LT风险及预后。在本研究中IPSS-R的重要参数骨髓原始细胞比例、IPSS-R细胞遗传学分组差和极差是MDS发生LT的独立危险因素，与既往文献报道一致[4],[10]–[11]。IPSS-R的其他参数，如血细胞减少水平和外周血细胞减少系数，在本研究LT组和未发生LT组中未见明显差异并对MDS发生LT无显著影响，这可能与纳入患者不同、分析方法有差异有关。既往研究[12]提示输血依赖是MDS发生LT风险的另一重要因素。由于本研究中患者就诊的当地医院输血标准尚不统一、血制品供应情况不一致、患者经济条件不等、身体代偿能力不同导致患者输血需求差异较大，因此未对患者输血依赖情况进行研究。

近年随着对MDS细胞遗传学和分子生物学研究的深入，细胞遗传学改变和基因突变对MDS患者LT的影响受到关注。我们研究表明，DNMT3A是初诊MDS LT的独立危险因素。与既往文献报道伴DNMT3A突变的初诊MDS患者发生LT风险增加的结果一致[13]–[14]。近年研究还发现DNMT3A不同突变位点和伴随不同突变对LT的作用也有一定区别，比如 DNMT3A R882位点突变相对于非R882位点突变LT风险增加，仅有DNMT3A突变的MDS患者相对于DNMT3A突变伴有SF3B1的患者LT风险更高[14]–[15]。但本研究中样本量有限，需纳入更多样本进一步研究。此外，Makishima等[16]通过发现相较于高危MDS，继发性急性髓系白血病（sAML）中FLT3、PTPN11、WT1、IDH1、NPM1、IDH2和NRAS基因突变高度富集，提出的这7个基因（1型突变）提示MDS发生LT风险增加。本研究中NPM1突变也是MDS发生LT的另一独立危险因素，NPM1是1型突变，此外，本研究结果与既往研究中NPM1突变在原始细胞<20％的髓系肿瘤性疾病中提示高风险向急性白血病转化的结论与本研究结果相一致[17]–[18]。

其他研究中TP53、IDH2、SRSF2等突变也提示高风险LT[19]–[22]。本研究单因素分析提示TP53和NRAS突变与MDS发生LT有关，多因素分析提示其对MDS发生LT无显著影响。与之前研究不完全一致，这可能与纳入人群、诊断分型、细胞遗传学等因素不同有关。SF3B1突变与MDS患者低白血病转化和良好预后相关，但本研究中SF3B1突变在LT组与未发生LT组差异无统计学意义，单因素分析SF3B1突变并非LT的影响因素[23]。

既往文献报道显示MDS发生LT过程中突变数量、突变负荷增加[8],[16],[24]–[25]，本研究中，6例MDS患者LT后4例患者获得至少1个新的基因突变，中位基因突变数目有增多趋势，VAF显著增加。Lindsley等[25]和Kim等[24]研究显示LT时新获得突变主要涉及转录因子和信号传导基因，因此当MDS患者出现新的转录因子和信号传导突变时需警惕LT可能。由于本研究中具有连续样本的病例数较少，未能进行克隆演化分析，待扩大样本量后进一步分析。

综上，本研究结果显示，MDS发生LT时，克隆复杂性（突变数目、突变负荷）呈增加趋势。骨髓原始细胞比例高、IPSS-R细胞遗传学分组差和极差、DNMT3A突变、NPM1突变提示MDS发生LT风险增高，我们需要加强对伴有这些危险因素的MDS患者的监测。但需注意MDS是一种异质性疾病，克隆演化是一个复杂的过程，因此需要继续深入研究LT发生机制，以便更精确地识别高危MDS患者。本研究存在以下不足：①作为单中心、回顾性研究，不可避免存在偏倚，造成结果误差。②具有连续标本的患者例数较少，未能对克隆演化进行统计学分析。③因本研究患者接受的同一药物的药物剂量或疗程、用药依从性差异较大，因此未对患者的治疗方案对MDS发生LT的影响进行分析；本研究结论有待全国多中心、前瞻性、更大的患者群体对研究结果进行证实。
